# Can Patient Reported Outcomes (PROs) from Population Surveys Provide Accurate Estimates of Pre-Admission Health Status of Emergency Hospital Admissions?

**DOI:** 10.2147/PROM.S215513

**Published:** 2020-02-11

**Authors:** Esther Kwong, Gary Abel, Nick Black

**Affiliations:** 1 Department of Health Services Research & Policy, London School of Hygiene and Tropical Medicine, London WC1H 9SH, UK; 2 College of Medicine and Health, University of Exeter, Exeter EX4 4PY, UK

**Keywords:** patient﻿-reported outcome measures, health status, health-related quality of life, self- report, retrospective, hospital admission, general population patient survey

## Abstract

**Introduction:**

The use of PROs for assessing the outcomes of emergency hospital admissions requires a means of estimating patients’ pre-admission health status. A possible alternative to asking patients to recall how their health was before the incident causing admission is to use estimates derived from matched samples from population surveys. Our aims were to explore the impact of different methods of matching and to compare the results with estimates based on retrospective reporting.

**Methods:**

First, elective hip arthroplasty patients were matched to respondents to the General Practice Patient Survey using age, sex, socio-economic status and number of comorbidities. The impact of restricting matching for locality and specific co-morbidities was explored. Second, the best matching method was applied to emergency admissions for laparotomy and for percutaneous coronary intervention (PCI) after acute myocardial infarction. Data were stratified by patient characteristics. Differences in mean EQ-5D scores between the patients and matched population respondents were tested using t tests.

**Results:**

Modifying the most basic form of matching by also taking locality and the specific comorbid conditions into account made no significant difference to the mean EQ-5D score for hip arthroplasty patients. Even using the most detailed matching possible, patients’ mean EQ-5D score was significantly different to that of the general population for all three cohorts. The difference was greatest for elective hip arthroplasty (0.22 v 0.64), less so for emergency laparotomy (0.56 v 0.72) and least for PCI (0.79 v 0.71). This reflects hip arthroplasty patients having a long-standing condition characterised by pain and limited mobility, whereas the other two cohorts may have enjoyed reasonable health until an unexpected acute episode led to their emergency admission.

**Conclusion:**

Routine PRO data acquired from population surveys cannot be used as an accurate alternative to retrospectively reported PROMs by patients during their emergency admission episode.

## Introduction

Patient-Reported Outcomes (PROs) have the potential to enhance the clinical management of patients and to assess the quality of providers’ performance.^1^,^2^ To date, use of PROs in assessing the outcome of hospital admissions has been largely restricted in elective surgery where before and after measurements of patients’ symptoms, functional status and health-related quality of life can be compared. The challenge for use in emergency admissions is how to measure a patient’s health status prior to an unexpected incident that caused an emergency admission.

It is not feasible to collect a pre-admission PRO but the use of retrospective or recalled assessment by patients has been established as a reliable alternative method. Studies of elective admissions have shown that retrospective PRO scores have high agreement with scores collected from patients before admission. Strong associations were found between retrospective and contemporary PROs in 21 out of 30 comparisons (correlation coefficients over 0.68) and 20 of 24 showed strong agreement (intraclass correlations over 0.75). A further study demonstrated the feasibility of using retrospective PROs in the NHS in England. That study found strong agreement between retrospective and contemporary disease-specific PROs and EQ-5D, with intra-class correlation coefficients of 0.8 for the disease-specific PROs (Oxford Hip Score and Oxford Knee Score), and 0.6 for the EQ-5D.^3^,^4^

An alternative approach that has been suggested is to derive expected PRO scores from respondents to general population surveys. Nine of the ten studies that have compared retrospective and population PRO scores have been conducted with trauma patients. Seven studies reported that, on average, patients recalled their health status as being better than that derived from age-sex-matched population samples^5^–^11^ while three reported no difference.^12^–^14^ These findings probably reflect that many trauma cases are from road traffic accidents among relatively healthy young adults. The only study that has considered non-trauma patients (intensive care unit patients with acute lung injury) reported their recalled health status was, on average, worse than that derived from population norms.^15^

Although the use of population norms has cost advantages, including less patient and staff burden of data collection, there is uncertainty as to whether it would provide a relevant and accurate assessment of pre-admission health status for groups of patients admitted to hospitals. It may be that with more extensive matching techniques, meaningful estimations of baseline health are possible for certain groups of patients, conditions and diseases. It is plausible that the baseline health status of patients admitted with sudden onset unexpected emergencies may have greater similarity with those in the population than is true for trauma admissions.

In this study, we make use of data from the English General Practice Patient Survey (GPPS), which included the EQ-5D instrument between 2011 and 2017 along with basic demographic information and self-reported co-morbidities.^16^ This rich population-based dataset offers the possibility of matching for several patient characteristics.

Our first aim was to explore the benefits of matching by comparing retrospective self-reported health status (mean EQ-5D scores) of hip arthroplasty patients with that of the general population of England. The second aim was to test the benefits of different matching techniques. The third aim was to test the impact of the optimum matching method by comparing mean differences between population EQ-5D scores and those reported retrospectively by patients admitted for elective hip arthroplasty, emergency laparotomy and acute myocardial infarction.

## Methods

Patients who participated in one of three cohort studies (receiving either an elective hip arthroplasty, emergency laparotomy for gastro-intestinal conditions (excluding appendicitis) or emergency percutaneous coronary intervention (PCI) after a myocardial infarction) were matched to GPPS respondents using several patient characteristics. Each patient was matched to as many GPPS respondents as fitted the matching criteria. The mean GPPS EQ-5D score for all those matched to a patient was used to compare with the patient’s retrospectively reported EQ-5D.

### Population Sample from GPPS

Data from the 2011–2012 GPPS (held at the University of Exeter) included the EQ-5D-3L, the same version as that used for the patient cohort. Questionnaires were sent in two waves, July 2011 and January 2012, to approximately 1.40 and 1.36 million patients, respectively. Non-responders were mailed a reminder in each of the two months following the initial questionnaire. Of the 2.76 million patients surveyed, 38% responded resulting in a sample of 1,037,946. Patients sent the GPPS comprise a stratified random sample of all adults registered with a general practice. Full details of the survey and its development are published elsewhere.^17^

Alongside patient experience items, patients were asked to report any long-standing health condition from a list of twelve common conditions: angina or long-term heart problem, arthritis or long-term joint problem, asthma or long-term chest problem, cancer in the last 5 years, deafness or severe hearing impairment, diabetes, epilepsy, high blood pressure, kidney or liver disease, long-term back problem, long-term mental health problem, long-term neurological problem and “another” long-term condition.^18^ They also reported age (18–24, 25–34, 35–44, 45–54, 55–64, 65–74, 75–84, and 85+ years) and sex. Further, their postcode of residence was used to assign a measure of socio-economic status, the Index of Multiple Deprivation (IMD).

### EQ-5D

The EQ-5D-3L is a generic PRO on a three-level ordinal scale (no problems, moderate problems and severe problems) for each of its five dimensions (mobility, self-care, usual activities, pain/discomfort and anxiety/depression).^19^ UK tariffs of utility were used to obtain an index score which ranges from −0.59 (the worst possible health state) to 1 (indicates best possible health state). The value of 0 is equal to death and negative values represent health states worse than death.^20^

### Patient Cohorts: Hip Arthroplasty, Emergency Laparotomy and PCI

EQ-5D from patients (n= 244) who had undergone hip arthroplasty (primary operation or revision surgery) in one of four NHS hospitals reported their pre-operative health status retrospectively in the immediate post-operative period prior to their discharge from hospital (Health Research Authority ethics approval was obtained from North East - Newcastle & North Tyneside 2 Research Ethics Committee (REC Ref: 16/NE/0081)).^4^ The mean EQ-5D score of the cohort was similar to that for all patients’ in a national audit in England. Information on comorbidity had previously been collected in a pre-operative questionnaire and covered: heart disease (for example, angina, heart attack or heart failure), high blood pressure, problems caused by a stroke, leg pain due to poor circulation, lung disease, diabetes, kidney disease, liver disease, cancer (within in the last 5 years), diseases of the nervous system (for example, Parkinson’s disease or multiple sclerosis), depression.

Emergency laparotomy patients (n= 261) and PCI patients (n= 396) were recruited from 11 and five NHS hospitals, respectively. Patients completed a retrospective questionnaire in the immediate period prior to their discharge from hospital following their emergency admission. The study received ethics approval from South East Coast - Brighton & Sussex Research Ethics Committee (REC reference: 16/LO/2053).^21^ The questionnaire included the same question about comorbidity as used for the hip arthroplasty study.

### Matching Patients to Population Sample

Patients were matched to GPPS population on sex, age, socioeconomic status and number of co-morbidities. The sample sizes were large enough, relative to the number of matching characteristics, to permit exact one-to-many matching. Age was categorised: 18–24, 25–34, 35–44, 45–54, 55–64, 65–74, 75–84, and 85+ years. Socioeconomic status (SES) was derived from the Index of Multiple Deprivation (IMD) of a patient’s local area (LSOAs) based on postcode, which was then grouped into quintiles based on the national ranking of areas by IMD to match the GPPS variable.^22^ Co-morbid conditions reported in the patient cohorts were mapped to the categories collected in the GPPS (Table 1).

**Table 1:** Co-Morbidities Reported in Patient and in the GPPS Questionnaires

Patient Questionnaires	GPPS Questionnaire
Heart disease	Angina or long-term heart problem
Arthritis	Arthritis or long-term joint problem
Lung disease	Asthma or long-term chest problem
Cancer	Cancer in the last 5 years
Diabetes	Diabetes
High blood pressure	High blood pressure
Kidney or Liver disease	Kidney or liver disease
Depression	Long-term mental health problem
Nervous system	Long-term neurological problem

Patients were matched to GPPS using personal characteristics available in both datasets. One-to-many matching was conducted, with one patient matched to as many GPPS respondents as fitted the matching criteria. The mean EQ-5D score of all GPPS respondents matched to a patient was used in the comparison between patients’ reported EQ-5D and GPPS EQ-5D. Mean EQ-5D scores for patients and for the population were compared.

First, exploratory matching was conducted with hip arthroplasty patients to determine whether the specific way of matching would change the differences between population and patients’ mean EQ-5D scores. Matching for age, sex, SES and number of comorbidities was compared with (i) restricting matching to GPPS respondents living in the same local authority and (ii) matching patients on the basis of specific combinations of co-morbidities. Data were stratified by patient characteristics and t tests were carried out to compare differences between patients’ EQ-5D means and population EQ-5D means by patient characteristics. As is customary, a p value of less than 0.05 was deemed statistically significant.

Second, influenced by the findings of the first phase, analysis of the emergency laparotomy and PCI patients was conducted. Similarly, data were stratified by patient characteristics and t tests were carried out to compare differences between patients’ EQ-5D means and population EQ-5D means by patient characteristics.

Despite the left skew of the EQ-5D data, we opted to use the paired *t*-test for comparisons between the three patient groups and population for three reasons. First, it enabled preservation of consistency in our comparisons between all the comparators. Second, the sample sizes satisfied guidelines for using parametric comparisons. And third, the *t*-test does not require the assumption of equal dispersion (equal variance) in the data when comparing between groups.

## Results

### Comparison of Matching Methods with Hip Arthroplasty Patients

Of 244 hip arthroplasty patients (80 men with mean age of 66 (range 35–90); 160 women with a mean age of 69 (range 29–90)). 25 were excluded because of missing data: 4 incomplete EQ-5D; 20 missing co-morbidities; one missing data on SES. Analyses were conducted with three different matching strategies:

#### Matched for Age, Sex, SES and Number of Comorbidities

The median number of matches per patient was 2434 (range 0–8052) though three patients could not be matched. The difference in EQ-5D scores between patients and the population was large (between 0.26 and 0.40) across all categories of patient characteristics.

#### Matched for Age, Sex, SES, Number of Comorbidities and Local Authority

**Figure 1:**
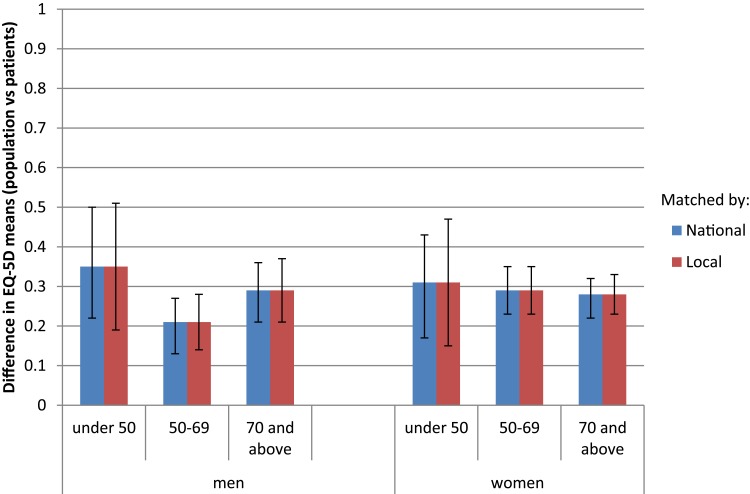
Impact of matching by local authority: comparison of difference in mean EQ-5D between hip arthroplasty patients and population reports (by age and sex).

The median number of matches per patient was 241 (range 0–1305); 17 patients could not be matched. The difference in mean EQ-5D between patients and the population was the same when matching was restricted to the same local authority and remained highly statistically significant (p<0.001) (Figure 1). Differences still ranged from 0.21–0.35 for different age, sex and SES categories (not shown).

#### Matched for Age, Sex, SES and Specific Comorbidities

**Figure 2:**
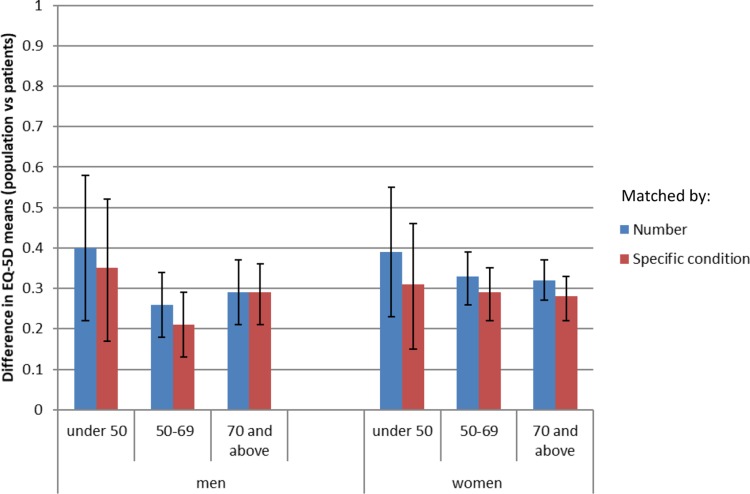
Impact of matching by specific comorbid condition: comparison of difference in mean EQ-5D between hip arthroplasty patients and population reports (by age and sex).

The median number of matches per patient was 336 (range 0–9832); seven patients could not be matched. The matched population mean EQ-5D was 0.64 (SD 0.23) which was significantly higher than the patients’ mean of 0.22 (SD 0.35); difference 0.42 (CI 0.39–0.44; p<0.001) (Table 2). Matching for specific comorbidities did not change the extent of the differences between population and patients' EQ-5D scores overall compared with matching by number of co-morbidities (Figure 2). The difference for age and sex sub-groups ranged from 0.21–0.35 using specific comorbidities compared with 0.26–0.40 using the number of conditions.

**Table 2:** Comparison of Mean EQ-5D (95% CI) of Hip Arthroplasty Patients and Population (Matched for Age, Sex, SES, and Specific Comorbidities)

Patient Characteristic	Patients		Population		Difference in Means (95% CI)
	Number	Mean EQ-5D (SD)	Number	Mean EQ-5D (SD)	
Overall	240	0.22 (0.35)	178,691	0.64 (0.24)	0.42 (0.39–0.44)
Sex					
Male	80	0.29 (0.35)	56,096	0.55 (0.23)	0.26 (0.21–0.31)
Female	160	0.19 (0.34)	122,632	0.48 (0.24)	0.29 (0.25–0.33)
Age					
49 or under	18	0.23 (0.36)	20,186	0.56 (0.25)	0.33 (0.21–0.45)
50–69	101	0.23 (0.36)	88,896	0.50 (0.24)	0.26 (0.21–0.31)
70 and above	121	0.21 (0.33)	69,603	0.50 (0.22)	0.29 (0.25–0.32)
SES					
1	21	0.28 (0.34)	16,536	0.57 (0.20)	0.29 (0.21–0.37)
2	42	0.20 (0.38)	38,456	0.51 (0.22)	0.31 (0.24–0.38)
3	65	0.25 (0.36)	54,212	0.54 (0.22)	0.29 (0.27–0.34)
4	62	0.27 (0.33)	36,468	0.48 (0.23)	0.21 (0.15–0.26)
5	49	0.13 (0.33)	33,019	0.47 (0.26)	0.34 (0.27–0.41)
missing	1				
Comorbidities					
0	22	0.24 (0.38)	72,160	0.73 (0.19)	0.49 (0.41–0.57)
1	79	0.29 (0.35)	76,675	0.62 (0.23)	0.33 (0.28–0.38)
2	70	0.23 (0.34)	23,463	0.51 (0.25)	0.28 (0.22–0.34)
3 or more	49	0.11 (0.34)	6393	0.42 (0.26)	0.31 (0.24–0.38)
missing	20

### Comparisons of Patients’ and Population EQ-5D for Three Patient Groups

Of 261 emergency laparotomy patients (121 men with mean age of 61 (range 18–90); 131 women with a mean age of 62 (range 21–91)). Nine were missing a complete baseline EQ-5D score, 19 were missing SES and four were missing co-morbidities. The median number of matches was 250 (range 0–11,421); five patients could not be matched. Of 396 PCI patients (305 men with mean age of 61 (range 27–92); 85 women with a mean age of 66 (range 44–94)). Six were missing a complete baseline EQ-5D score, 23 were missing SES, and one was missing comorbidities. The median number of matches 139 (range 0–11,541); six could not be matched.

The significant difference between hip arthroplasty patients’ mean EQ-5D and that of a matched population already reported (0.42; CI 0.39–0.44) (Table 2) was also observed for emergency laparotomy patients, although the size of the difference was smaller (0.13; CI 0.10–0.15, p<0.001) (Table 3). While the difference for PCI patients was also statistically significant, the direction of difference was reversed with the patients reporting better health than the matched population (−0.09; Cl −0.12 to −0.07, p<0.001) (Table 4).

**Table 3:** Comparison of Mean EQ-5D (95% CI) of Emergency Laparotomy Patients and Population (Matched for Age, Sex, SES, Local Authority and Specific Comorbidities)

Patient Characteristic	Patients		Population		Difference in Means (95% CI)
	Number	Mean EQ-5D (SD)	Number	Mean EQ-5D (SD)	
Overall	252	0.56 (0.40)	374,519	0.72 (0.22)	0.13 (0.10–0.15)
Sex					
Male	121	0.62 (0.35)	152,375	0.66 (0.22)	0.04 (0.001–0.08)
Female	131	0.50 (0.43)	219,555	0.65 (0.23)	0.15 (0.11–0.18)
Age					
49 or under	62	0.48 (0.46)	186,329	0.72 (0.20)	0.24 (0.19–0.29)
50–69	94	0.52 (0.39)	120,612	0.63 (0.24)	0.11 (0.06–0.16)
70 and above	96	0.66 (0.33)	64,989	0.64 (0.23)	−0.02 (−0.1–0.06) p=0.62*
SES					
1	37	0.57 (0.39)	86,250	0.79 (0.21)	0.22 (0.15–0.28)
2	45	0.61 (0.43)	51,128	0.73 (0.21)	0.10 (0.04–0.15)
3	47	0.66 (0.31)	93,699	0.75 (0.21)	0.09 (0.03–0.14)
4	51	0.53 (0.37)	60,014	0.66 (0.25)	0.13 (0.06–0.19)
5	53	0.45 (0.42)	77,103	0.58 (0.27)	0.13 (0.06–0.20)
missing	19				
Comorbidities					
0	56	0.66 (0.39)	299,449	0.81 (0.16)	0.15 (0.11–0.19)
1	80	0.60 (0.39)	59,163	0.74 (0.23)	0.14 (0.09–0.19)
2	50	0.53 (0.39)	8988	0.60 (0.26)	0.07 (0.002–0.14)
3 or more	62	0.46 (0.39)	4330	0.41 (0.26)	−0.05 (−0.1–0.02) p=0.17*
missing	4				

**Note:** *No significant difference between patients and population.

**Table 4:** Comparison of Mean EQ-5D (95% CI) of PCI Patients and Population (Matched for Age, Sex, SES, Local Authority and Specific Comorbidities)

Patient Characteristic	Patients		Population		Difference in Means (95% CI)
	Number	Mean EQ-5D (SD)	Number	Mean EQ-5D (SD)	
Overall	390	0.79 (0.28)	411,388	0.71 (0.23)	−0.09 (−0.12–0.07)
Sex					
Male	305	0.81 (0.28)	339,012	0.67(0.22)	−0.14 (−0.16–0.12)
Female	85	0.74 (0.29)	72,376	0.59 (0.26)	−0.15 (−0.21–0.09)
Age					
49 or under	60	0.76 (0.36)	90,926	0.64 (0.21)	−0.12 (−0.17–0.07)
50–69	219	0.80 (0.28)	270,628	0.67 (0.23)	−0.13 (−0.16–0.10)
70 and above	111	0.80 (0.23)	49,834	0.68 (0.24)	−0.13 (−0.17–0.09)
SES					
1	69	0.85 (0.20)	90,276	0.72 (0.20)	−0.13 (−0.18–0.08)
2	60	0.83 (0.22)	96,405	0.75 (0.23)	−0.08 (−0.14–0.02)
3	90	0.79 (0.23)	81,817	0.69(0.23)	−0.10 (−0.15–0.05)
4	93	0.73 (0.37)	87,157	0.65(0.24)	−0.08 (−0.13–0.03)
5	55	0.77 (0.34)	47,536	0.61 (0.27)	−0.16 (−0.23–0.09)
missing	23				
Comorbidities					
0	57	0.90 (0.16)	311,228	0.83 (0.17)	−0.07(−0.11–0.03)
1	109	0.88 (0.18)	75,761	0.75 (0.20)	−0.013(−0.17–0.09)
2	94	0.81 (0.26)	17,722	0.71(0.24)	−0.10 (−0.15–0.05)
3 or more	129	0.66 (0.35)	6677	0.45 (0.26)	−0.21 (−0.28–0.14)
missing	1				

When stratified by patient characteristics, the mean differences of patients’ EQ-5D from that of their matched populations were significantly different across nearly all stratified groups for all three patient groups. With the exception of PCI patients, patients’ reported a lower baseline EQ-5D than that for the matched population. The only categories for which there was no significant difference were for emergency laparotomy patients over 70 years of age, and with 3+ co-morbidities (Table 3).

## Discussion

### Main Findings

Modifying the most basic form of matching (using the whole population adjusted for sex, age, SES and number of comorbidities) by also taking locality and the specific comorbid conditions into account made no substantial difference to the estimated EQ-5D mean score. Given the larger sample available when using national data, matching using the whole population is the preferred option.

Despite the use of specific comorbidities conferring no benefit over a simple count from the exploratory matching with hip patients, the former was chosen for comparing differences between patients and the population as co-morbidity has been shown to influence the health status of respondents in the GPPS in prior published research.^23^

Patients’ mean EQ-5D score was significantly different than that of the general population for all three cohorts. The difference was greatest for elective hip arthroplasty patients (0.22 v 0.64), less for emergency laparotomy (0.56 v 0.72) and least for PCI (0.79 v 0.71) in whom the direction of difference was reversed with patients reporting higher baseline EQ-5D than the population. This corresponds to the clinical context in which hip arthroplasty patients have a long-standing condition characterised by pain and limited mobility, whereas the other two cohorts of emergency patients may have enjoyed reasonable health until an unexpected acute episode led to their emergency admission. This is particularly true for the PCI patients, many of whom had no prior symptoms making it plausible they had better health status than those in the matched population of respondents to the GPPS. The only sub-groups in whom their self-reported EQ-5D did not differ significantly from that of the matched population were those emergency laparotomy patients who were least healthy (aged over 70 years, and with two or more comorbidities).

Our findings differ from most published studies which have reported general populations being healthier than patients.^5^–^7^,^9^–^11^ This reflects that most of those studies were limited to trauma patients rather than patients with long-term illnesses or conditions.^5^–^7^,^9^–^14^ The one exception was a study of medical inpatients which reported similar findings to our study.^15^ Thus, our findings are consistent with the suggestions by other authors that the acute injury population may be healthier, whereas patients with medical and surgical needs are less healthy compared to the general population.

These findings confirm that routine PRO data acquired from population surveys cannot be used as an accurate alternative to retrospectively reported PROs by medical and surgical patients during their emergency admission episode.

### Strengths and Limitations

There are three limitations to consider. First, the validity of the GPPS data. Although it is a large national survey, the response rate in 2011–2012 was only 38%, albeit similar to that achieved in other surveys using a similar methodology.^17^ In addition, co-morbidity data were missing for 13% and EQ-5D scores were incomplete for 20%.^23^ Responders are more likely to be women, middle-aged and those in affluent areas, factors that will influence the mean EQ-5D score. However, given that in this study the data were matched for sex, age and SES, any response bias will be limited to any other characteristics such as ethnic group or educational attainment or indeed health status itself.^24^ Although it is not possible to estimate the impact of bias due to these variables, published meta-analyses on probability sampled surveys suggest that response rates are not a strong predictor for response bias.^25^

Second, comorbidity data in both the retrospective patient cohorts and the GPPS are based on respondents’ reports. In both samples, it is possible that respondents might under or over-report conditions. However, previous studies suggest that the incidence of comorbidities reported by patients is similar to that from medical records except for diabetes, high blood pressure and long-term back problems.^23^

Finally, the stratified analysis used may not have been adequate to control for confounding as EQ-5D is age-dependent; however, cross tabulation of stratified age (ten-year bands) of EQ-5D scores revealed no significant associations with sex or SES.

### Implications for Policy

These exploratory findings show that use of the GPPS is not suitable for use in place of a retrospective PRO in three particular patient groups. Population and groups of patients remain significantly different even with specific matching and only in certain sub-segments of the population were they similar. It would, therefore, not be appropriate to assess the outcome of care for those admitted as emergencies by comparing their PRO scores with that derived from the general population. In the situation of hip arthroplasty and EL patients, given that before their admission the health status of these patients is, on average, significantly worse than their matched peers in the population, it is not reasonable to expect that they will attain the mean level of health status of the population. To assume that may suggest that the care they receive both during their emergency admission and subsequently in the community is sub-optimal. The reverse is true of PCI patients. Thus the use of PROs in emergency admissions needs to incorporate retrospectively collected PROs. The challenge is how this can be done routinely in a cost-effective way.
